# DEC2–E4BP4 Heterodimer Represses the Transcriptional Enhancer Activity of the EE Element in the Per2 Promoter

**DOI:** 10.3389/fneur.2015.00166

**Published:** 2015-07-23

**Authors:** Shintaro Tanoue, Katsumi Fujimoto, Jihwan Myung, Fumiyuki Hatanaka, Yukio Kato, Toru Takumi

**Affiliations:** ^1^Graduate School of Biomedical Sciences, Hiroshima University, Hiroshima, Japan; ^2^RIKEN Brain Science Institute, Wako, Saitama, Japan; ^3^CREST, Japan Science and Technology Agency, Tokyo, Japan

**Keywords:** PER2, DEC2, E4BP4, circadian rhythm, E-box

## Abstract

The circadian oscillation of clock gene expression in mammals is based on the interconnected transcriptional/translational feedback loops of *Period (Per)* and *Bmal1*. The *Per* feedback loop initiates transcription through direct binding of the BMAL1–CLOCK (NPAS2) heterodimer to the E-box of the *Per2* promoter region. Negative feedback of PER protein on this promoter subsequently represses transcription. Other circadian transcription regulators, particularly E4BP4 and DEC2, regulate the amplitude and phase of *Per2* expression rhythms. Moreover, a direct repeat of E-box-like (EE) elements in the *Per2* promoter is required for its cell-autonomous circadian rhythm. However, the detailed mechanism for repression of the two core sequences of the EE element in the *Per2* promoter region is unknown. Here, we show that E4BP4 binds to the *Per2* EE element with DEC2 to repress transcription and identify the DEC2–E4BP4 heterodimer as a key repressor of the tightly interlocked *Per2* feedback loop in the mammalian circadian oscillator. Our results suggest an additional modulatory mechanism for tuning of the phase of cell-autonomous *Per2* gene expression cycling.

## Introduction

The circadian clock regulates many physiological and behavioral cues, such as hormone secretion, metabolism, body temperature, immune response, and sleep–wake rhythms (1–3). Critical to these processes, the mammalian transcriptional regulators PERIOD (PER), cryptochrome (CRY), BMAL1, and NPAS2/CLOCK play core roles in sustaining interlocked circadian rhythms (4). In particular, BMAL1 forms a heterodimer with either NPAS2 or CLOCK to activate the *Per* promoter (5, 6). PER and CRY (CRY1 and CRY2), meanwhile, function in negative feedback loops that repress BMAL1–NPAS2/CLOCK, thereby completing a transcription/translation-based autoregulatory feedback loop (7, 8). Three mammalian orthologs of *Per*, named *Per1*, *Per2*, and *Per3*, have been previously identified (9–14). PER1 and PER2 play important roles for circadian oscillation in the master clock suprachiasmatic nucleus (SCN), whereas PER3 participates in timekeeping in peripheral tissues, such as pituitary and lung (15). *Per2* knockout mice exhibit reduction of *Per1* and *Clock* expression levels (16–18), indicating PER2 plays a prominent role in regulating expression of circadian clock genes. *Per2* expression mechanisms are different from the classical mechanisms of circadian gene expression in the sense that an E-box-like (EE) (CACGTT) sequence, rather a canonical E-box consensus (CACGTG) sequence, in the *Per2* promoter is essential for robust circadian transcription of *Per2* (19). The two core sequences of the EE-element in the *Per2* promoter region, CACGTT and TATGTG, named E1 and E2, respectively, are required for *Per2* rhythmic expressions (20). The E1 site-binding transcription regulators are BMAL1 and CLOCK, while the E2 site-binding transcription regulators remain unidentified (20).

E4BP4, a basic leucine zipper (bZIP) transcription factor, and DEC1/2, basic helix–loop–helix (bHLH) transcription factors, modulate amplitude and phase of *Per*1/2 expression rhythms (21, 22). Furthermore, *Dec1* and *Dec2* are expressed in the SCN abundantly. DEC1 represses the CACGTG E-box weakly, whereas DEC2 represses the same E-box more strongly (23). *Dec*1/2 knockout mice lack normal resetting for phase shift of light–dark cycles and exhibit abnormal amplitude of *Per2* expression rhythms in the SCN (22). DEC2 forms a heterodimer with multiple proteins, such as MyoD and retinoid X receptor (RXR), to modulate a wider variety of gene expressions (24, 25). It is thus expected that DEC2 forms heterodimers with hitherto unknown transcription factors to bind the *Per2* EE-element and fine-tune the phase of circadian oscillation.

Physiological roles of E4BP4 have been studied in the chicken pineal gland. The light pulse at early subjective night induces *E4bp4* expression in the pineal gland, which acts as a light-dependent suppressor of *Per2* expression (26, 27). In mammals, *E4bp4* is expressed in the SCN in a circadian manner by which the peak of *E4bp4* expression occurs at night (28). The E4BP4 binding site (B-site) has been identified, and E4BP4 represses *Per2* promoter activity by binding to this B-site, rather than the *Per2* EE-element (29). However, E4BP4 regulates the mouse *Per2* promoter region (−105 ~ +110), which includes the EE-element but not the B-site (19). E4BP4 may bind to this *Per2* promoter region through a binding mechanism different from that of the B-site binding.

In the present study, we investigated how DEC2 and E4BP4 bind to the *Per2* EE-element to modulate the phase of circadian oscillation. Here, we report DEC2 and E4BP4 form a heterodimer to repress *Per2* promoter activity by binding to the E2 consensus of the *Per2* EE-element. Our results suggest that the DEC2–E4BP4 heterodimer plays a crucial role in modulating phase of *Per2* circadian rhythms.

## Results

### DEC2 and E4BP4 form a heterodimer

It remains unknown how DEC2 regulates amplitude and phase of circadian rhythms. A homolog of DEC2, *Drosophila* clockwork orange (CWO), functions as both an activator and a repressor of clock gene expression (30). One possible mechanism for these functions of CWO has been proposed to be the binding of various unknown transcription regulators periodically to regulate circadian rhythms. In fact, DEC2 binds to various transcription regulators, namely BMAL1, MyoD, and RXR (23–25). We therefore hypothesized that DEC2 forms a heterodimer with an unknown transcription regulator to control the *Per2* EE-element-driven promoter activity.

**Figure 1:**
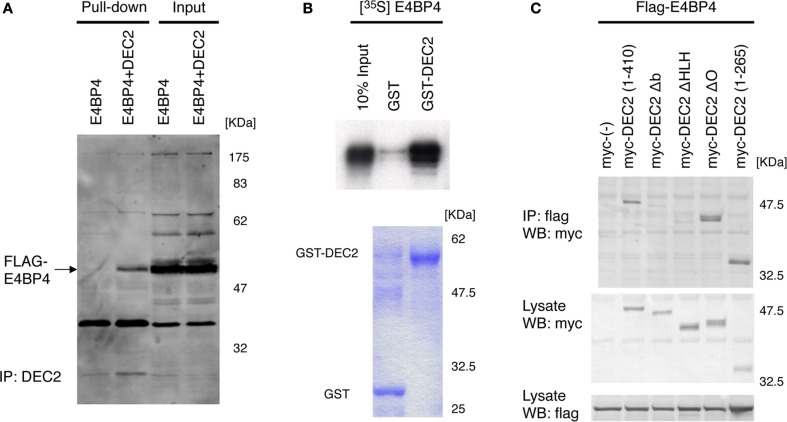
**DEC2 forms a heterodimer with E4BP4**. **(A,B)** Immunoprecipitation of E4BP4 and DEC2 complex. Flag-tagged E4BP4–DEC2 heterodimer was pulled down with anti-DEC2 antibody and protein A agarose beads. Flag-tagged E4BP4 protein was detected using immunoblotting with anti-FLAG antibody. Flag-tagged E4BP4–DEC2 heterodimer was detected in NIH3T3 cells co-expressing DEC2 and E4BP4 but not in the cells expressing E4BP4 only **(A)**. The data are a representative result of three independent experiments. Radioisotope labeled S^35^-E4BP4 was pulled down with GST-tagged DEC2 and glutathione-coupled agarose beads. The bottom panel shows the corresponding SDS-PAGE gel stained with Coomassie blue **(B)**. **(C)** bHLH domain of DEC2 plays a crucial role in binding of E4BP4. COS-7 cells were transfected with myc-tagged Dec2 expression vectors together with flag-tagged E4BP4 expression vector. Cell lysates were immunoprecipitated with anti-flag antibody and immunoblotted with anti-myc antibody. To confirm expression of E4BP4 and DEC2 proteins, aliquots of the total cell lysates were immunoblotted with the anti-flag and anti-myc antibodies. b, basic region; HLH, helix–loop–helix domain; O, orange domain. Numbers indicate the position of amino acids in the DEC2 protein.

DEC2 is speculated to have similar functions as E4BP4 for circadian rhythms. *Per2* expression levels are repressed by E4BP4 (19, 26, 29), suggesting that E4BP4 potentially modulates the amplitude and phase of circadian rhythms in a manner similar to that of DEC2. Based on this logic, we assessed how E4BP4 interacts with DEC2 to modulate circadian rhythms. Using immunoprecipitation and glutathione *S*-transferase (GST) pull-down assays, we discovered that DEC2 forms a heterodimer with E4BP4 (Figures 1A,B). Furthermore, given the previous finding that the DEC2 bHLH domain plays a crucial role in forming a heterodimer with MyoD (24), we assessed formations of heterodimer between various DEC2 fragments and E4BP4. DEC2 bHLH domain showed binding with high amount of E4BP4 in the co-immunoprecipitation experiment (Figure 1C). This result indicates that DEC2 bHLH is important for DEC2–E4BP4 heterodimer formation.

### DEC2–E4BP4 heterodimer binds to the E2 consensus of the *Per2* EE-element

**Figure 2:**
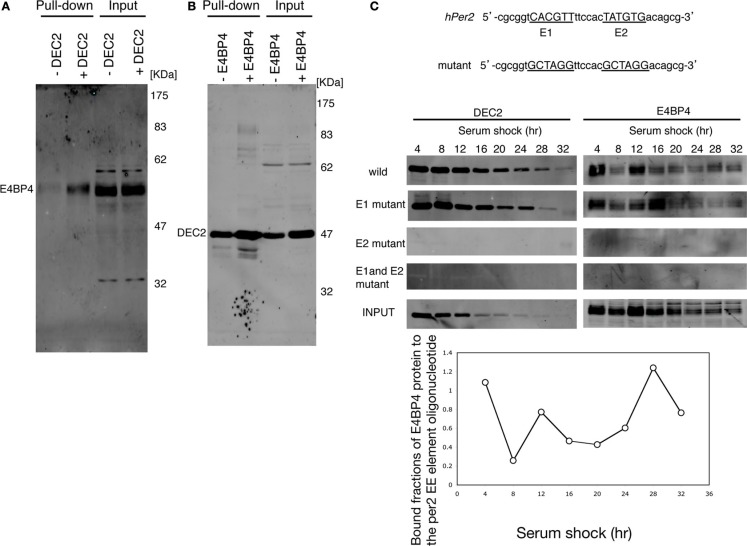
**E4BP4 binds to the E2 consensus of *Per2* EE element via DEC2**. **(A)** E4BP4 binds to oligonucleotide of the *Per2* EE element in the presence of DEC2. The complexes of biotin-labeled *Per2* EE-element probe (20) and E4BP4 protein from nuclear extracts of E4BP4-overexpressing NIH3T3 cells were pulled down using streptavidin beads. Detection of E4BP4 was performed using immunoblotting with anti-E4BP4 antibody. E4BP4 was pulled down in the nuclear extracts of NIH3T3 cells co-expressing DEC2 and E4BP4 (indicated with +DEC2). E4BP4 was not pulled down in the nuclear extracts of NIH3T3 cells expressing only E4BP4 (indicated with −DEC2). **(B)** DEC2 binds to oligonucleotide of the *Per2* EE element without E4BP4. Detection of DEC2 was performed using immunoblotting with anti-DEC2 antibody. DEC2 was pulled down in nuclear extracts of NIH3T3 cells both expressing only DEC2 and co-expressing DEC2 and E4BP4, indicated with −E4BP4 and +E4BP4, respectively. **(C)** DEC2–E4BP4 heterodimer binds the E2 consensus of *Per2* EE element. DEC2–E4BP4 heterodimer was pulled down from nuclear extracts of NIH3T3 cells co-expressing DEC2 and E4BP4 every 4-h after cyclohexylamine-shock. Detection of E4BP4 or DEC2 was performed using immunoblotting with DEC2 (left panel) and E4BP4 (right panel). Both DEC2 and E4BP4 were pulled down by wild-type and the E1 mutant probes but not by E2 mutant and E1-E2 mutant probes. Wild-type and mutant sequences of *Per2* EE element probe are shown in the upper panel. Representative results of three independent experiments are shown. The bottom figure shows the quantification of bound fractions of E4BP4 to the wild-type per EE element.

Since the *Per2* EE-element is essential for normal circadian oscillation (20), we assessed the possibility of DEC2–E4BP4 heterodimer binding to the *Per2* EE-element. E4BP4 bound to the oligonucleotide probe of the *Per2* EE-element in the presence of DEC2, whereas E4BP4 failed to bind to the *Per2* EE-element in the absence of DEC2 (Figure 2A). On the contrary, DEC2 bound to the *Per2* EE-element in the absence of E4BP4 (Figure 2B). These results indicate that DEC2 mediates E4BP4 binding to the *Per2* EE-element.

The *Per2* EE-element consists of two consensus sequences, named E1 and E2 (20). We tested whether the DEC2–E4BP4 heterodimer binds to E1 or to E2 through an oligonucleotide pull-down experiment. In *Drosophila*, circadian transcription regulators bind to the *Per* promoter dynamically (31). Therefore, we tested time-dependent DEC2 and E4BP4 binding to the *Per2* EE-element in serum shocked NIH3T3 cells. DEC2 and E4BP4, which were expressed in the nucleus of the serum shocked NIH3T3 cells, dynamically bound to the oligonucleotide probe with the mutant E1 and the wild-type E2 but failed to bind to the probe with the wild-type E1 and the mutant E2 (Figure 2C). This result indicates that the DEC2–E4BP4 heterodimer binds to the E2 consensus of the *Per2* EE-element and not to the E1 consensus sequence.

### DEC2–E4BP4 heterodimer represses promoter activity of the *Per2* EE-element region

**Figure 3:**
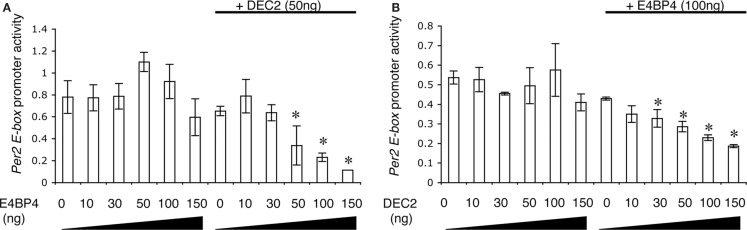
**DEC2 represses *Per2* EE element promoter activity**. **(A,B)** E4BP4–DEC2 heterodimer represses *Per2* EE element promoter activity. NIH3T3 cells were transfected with different amounts of *E4bp4*/pcDNA3.1 or *Dec2/*pcDNA3.1 plasmid vector, 100 ng *Per2* EE element luciferase/pGL3 plasmid vector, 3 ng of *Bmal1*/pcDNA3.1 and *Clock*/pcDNA3.1, and 20 ng pRL-TK plasmid vector. *Per2* EE element promoter activity is presented as relative values of the *Per2EE* promoter-driven luciferase activity compared to internal control luciferase activity (pRL-TK). E4BP4 repressed the *Per2* EE element-driven promoter activity in the presence of DEC2 **(A)**. DEC2 repressed the *Per2* EE element-driven promoter activity in the presence of E4BP4 **(B)**. Error bars represent SEM from three independent experiments. Asterisk (*), *p* < 0.05 versus 0 ng of E4BP4 or DEC2.

To define the effects of the DEC2–E4BP4 heterodimer on promoter activity of the *Per2* EE-element, we measured *Per2* EE-element region-driven luciferase activities in the presence of DEC2 and E4BP4. Promoter activities of the *Per2* EE-element region were not affected by single expressions of DEC2 or E4BP4, whereas co-expression of E4BP4 and DEC2 repressed promoter activities of the *Per2* EE-element (Figures 3A,B). Therefore, it is a heterodimer of DEC2 and E4BP4 that represses promoter activity of the *Per2* EE-element region. Furthermore, we quantified the ratio of binding between DEC2 and E4BP4 with exponential fitting. Half-maximal inhibition dosages (IC_50_) of DEC2 and E4BP4 with respective dimerization partners, E4BP4 100 ng and DEC2 50 g, were estimated to be 101.1 and 56.6 ng, respectively, indicating a 1:1 stoichiochemistry.

### Mathematical modeling shows DEC2–E4BP4 heterodimer has an impact on phase of *Per2* expression cycling

**Figure 4:**
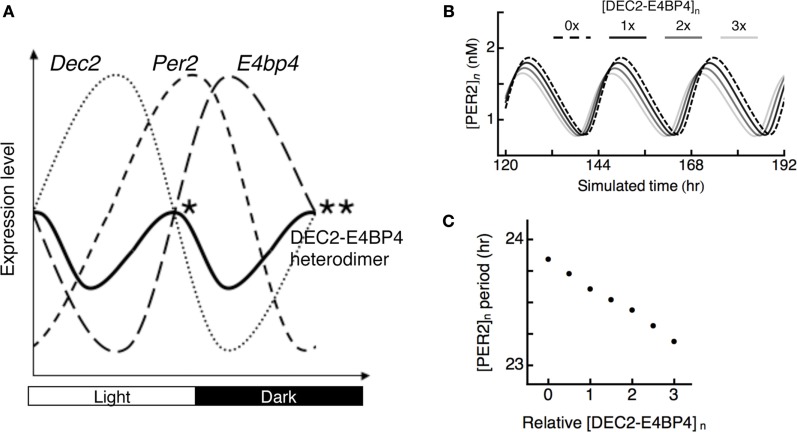
**Possible roles of DEC2–E4BP4 heterodimer in the interlocked *Per2* oscillation**. **(A)** Phases of clock gene cycling in SCN. *Dec2* expression cycling is opposite to *E4bp4* expression cycling in the SCN (23, 28). Two peaks of DEC2–E4BP4 heterodimer levels in the SCN should be at subjective dusk (*) and dawn (**), at which DEC2 and E4BP4 expression levels are equal. *Per2* expression levels are high at subjective dusk and trough at subjective dawn in SCN (11, 12). DEC2–E4BP4 heterodimer represses *Per2* expression levels to determine time points of peak and trough of *Per2* expression cycling. **(B)** Theoretical model of PER2 protein expression cycling under various amounts of DEC2–E4BP4 heterodimer. Oscillations of PER2 expression levels under various amounts of [DEC2–E4BP4]*_n_* (*n* = 0, 1, 2, 3) are indicated with dashed line (*n* = 0), black solid line (*n* = 1), gray solid line (*n* = 2), and light gray line (*n* = 3). The simulated nuclear concentration of DEC2–E4BP4 heterodimer ([DEC2–E4BP4]*_n_*) induces phase advance of PER2 ([PER2]*_n_*) expression cycling in a concentration-dependent manner. **(C)** Periods of the PER2 oscillations under various amount of DEC2–E4BP4 heterodimer in **(B)**. High concentration of DEC2–E4BP4 heterodimer shortens the period of PER2 oscillation.

Phases of DEC2 and E4BP4 are opposite in the SCN. Peak points of DEC2 and E4BP4 expression levels are at daytime and midnight (23, 28). Expression levels of DEC2–E4BP4 heterodimer should be high at both subjective dusk and dawn, at which DEC2 and E4BP4 expression levels are equal (Figure 4A). To evaluate the logical consequence of the DEC2–E4BP4 heterodimer repression in *Per2* expression cycling, we introduced a mathematical model of circadian oscillation. Since the kinetics of DEC2 and E4BP4 has not been considered in previous mathematical models, we created a hybrid model based on the Becker–Weimann model of the circadian clock (32) by inserting contributions of DEC2 and E4BP4 as exogenous sinusoidal variations as proposed earlier (33). DEC2–E4BP4 heterodimer concentration is given by multiplying the concentration of DEC2 and E4BP4, in accordance with our observation of 1:1 binding (Figure 3). High levels of DEC2–E4BP4-induced phase advances of *Per2* expression cycling (Figure 4B). Three times of the tentative norm of the DEC2–E4BP4 level induced an ~1-h phase advance of *Per2* expression cycling per day (Figure 4C). The hybrid model test the consequence of rhythmic repression, which shows that modulation of the DEC–E4BP4 heterodimer has an impact on *Per2* expression cycling.

## Discussion

Many circadian transcription regulators dynamically form complexes with other circadian transcription regulators for circadian timekeeping. Here, we found a new complex, the E4BP4–DEC2 heterodimer, which serves as repressor of *Per2* promoter activity. E4BP4 and DEC2 are thought to be negative circadian regulators independently (23, 29). In addition to previous reports, we found E4BP4 and DEC2 form a heterodimer to cooperatively regulate the clock function. E4BP4 and DEC2 play important roles in modulating the phase of circadian rhythms. Overexpressing E4BP4 shortens the period length of *Per2* oscillation (19). DEC2 is required for normal circadian phase resetting (22). E4BP4 and DEC2 modulate phase of *Per2* expression cycling cooperatively through formation of a heterodimer.

With respect to cycling phases of *Dec2, Per2*, and *E4bp4* expression levels, we speculate that two peaks of DEC2–E4BP4 heterodimer levels should be around subjective dusk and dawn, at which points *Dec2* and *E4bp4* expression levels are the same in the SCN (Figure 4A). As for the significance of these two peaks of E4BP4-heterodimer in *Per2* expression cycling, we propose the following model: at subjective dusk, the DEC2–E4BP4 heterodimer determines the peak time point of *Per2* expression levels through repression of *Per2* expression induction. At subjective dawn, the DEC2–E4BP4 heterodimer determines the trough point of *Per2* expression cycling through further encouraging the decrease of *Per2* expression. This dynamic binding eventually fine-tunes the phase of *Per2* expression cycling. This idea is supported by a previous report that showed the overexpression of E4BP4 induces a shorter period length for *Per2* oscillation (19). Furthermore, the mathematical model supports the idea that DEC2–E4BP4 modulates the phase of *Per2* expression cycling by shortening the length of period (Figures 4B,C), which is consistent with the recent report (34). Increasing DEC2–E4BP4 heterodimer enhances phase advances of *Per2* expression cycling in this model. Additionally, the mathematical model can explain the mechanism of phase advance of *Per2* expression cycling by light at early subjective night. Light induces E4BP4 expression in the avian circadian system (26). The induced E4BP4 forms a heterodimer with DEC2 and binds to the *Per2* EE-element to repress *Per2* promoter activities. The oscillatory acceleration through this suppression eventually creates phase advance of *Per2* expression cycling.

In this study, we showed that the E4BP4–DEC2 heterodimer binds to the E2 consensus to repress *Per2* promoter activity (Figures 2 and 3). Considering that E4BP4 and DEC2 are modulators of clock functions rather than drivers of clock functions, such as BMAL1 and CLOCK, we can propose that the E2 consensus is responsible for modulating the phase and amplitude of circadian oscillation. EE-elements are also located on other promoter regions of many BMAL1 target genes, such as *Dbp*, *Nr1d1*, and *Tef* (20, 35, 36). E4BP4–DEC2 heterodimer can thus regulate the expression rhythms of these genes as well, though further experiments are needed.

The dynamic binding of E4BP4 to the *Per2* EE-element we observed, in addition to single E4BP4 binding to the B-box in the *Per2* exon, likely contributes to controlling *Per2* expression (29). DEC2 is required for E4BP4 binding to the *Per2* EE-element. DEC2 is able to bind to various proteins, MyoD, RXR, histone deacetylase, and Sirtuin 2 (24). Understanding how a complex of DEC2 and these proteins regulates the *Per2* EE-element activities will shed light on mechanisms to modulate phases of circadian oscillators.

## Materials and Methods

### Plasmids

The bacterial expression vector for full-length *Dec2* that fused with GST was described previously (24). Mammalian expression vectors for myc-tagged full-length and deletion mutants of DEC2 were described previously (24).

### Cell culture and DNA transfection

NIH3T3 cells were cultured as described (37). DNA transfection was performed according to the manufacturer’s instructions (Lifetechnologies).

### Immunoprecipitation

One microgram of pcDNA3.1-*Dec2* (38) and pcDNA3.1-FLAG-*E4bp4* (19) were transfected into NIH3T3 cells. After 28 h, the cells were harvested and homogenized using ultrasonication in buffer containing 20 mM Tris-HCl (pH 7.5), 150 mM NaCl, 0.3% Triton X-100, 1 mM PMSF, 1 mg/ml leupeptin, and 1 mg/ml aprotinin. The homogenate was centrifugated (15,000 × *g*, 5 min, 4°C). Supernatants were incubated with Protein A agarose beads (Roche) and anti-DEC2 antibody (24) (dilution 1:200) for overnight, 4°C. The immunoprecipitated FLAG tagged E4BP4 was detected on Immunoblotting using anti-FLAG antibody (Sigma-Aldrich; dilution 1:500). The interactions of E4BP4 with myc-tagged DEC2 mutant proteins were also examined. The co-immunoprecipitation assay was performed according to a previously described protocol (24).

### GST-pull assay

The BL21 strain of *Escherichia coli* was transformed with plasmid pET-41-*Dec2* for expression of GST-DEC2 fusion protein or, as a control, pET-41 for expression of GST. GST-pull down assay was performed using purified GST or GST-DEC2 fusion proteins, as described previously (24). [^35^S] methionine-labeled proteins were synthesized *in vitro* using the TNT Quick Coupled Transcription/Translation System (Promega, WI, USA).

### Luciferase reporter assay

Different amounts of pcDNA3.1-*Dec2* and pcDNA3.1-FLAG-*E4bp4* were cotransfected with 20 ng pRL and 100 ng *Per2* EE-element promoter-driven luciferase (20) into NIH3T3T cells. Transfected cells were assayed for luciferase activity in a luminometer (Turner Designs, CA, USA) by using the dual-luciferase reporter assay system (Promega, WI, USA).

### Oligonucleotide probe pull-down experiment

Three micrograms of pcDNA3-*Dec2* and pcDNA3-*e4bp4* were transfected into NIH3T3T cells. At 23 h after transfection, the medium was exchanged for 100 nM cyclohexylamine containing medium, and 2 h later, the medium was replaced with 10% FBS containing medium. The cells were harvested every 4 h and used for oligonucleotide probe pull-down experiment as described (20). The pull-downed E4BP4 and DEC2 were detected on Immunoblot using anti-E4BP4 antibody (39, 40) and anti-DEC2 antibody.

### Mathematical modeling

An expected time course of the DEC2–E4BP4 heterodimer is inserted directly into as an inhibitory term in the Michaelis–Menten equation (33) describing *Per2* transcription (*y*_1_). The dynamic DEC2–E4BP4 heterodimer concentration is given by

$$n\times 0.55\left(\cos\left(\frac{2\pi t}{24}-7\right)+2.4\right)\cdot 0.4\left(\cos\left(\frac{2\pi t}{24}-10\right)+2.6\right)$$

In this equation, the first cosine describes the DEC2 time course, the second describes the E4BP4 time course, and *n* determines the relative concentration of the DEC2–E4BP4 heterodimer. We inserted this equation into the equations of the Becker–Weimann model (32) as follows:

$$\begin{array}{c} {\dot{y}}_{1}=\frac{\upsilon_{1b}\left(y_{7}\left(t\right)+c\right)}{k_{1\text{b}}\left(1+{\left(\frac{y_{3}\left(t\right)}{{k_{1}}_{\text{i}}}\right)}^{p}+\left(n\cdot 0.55\left(\cos\left(\frac{2\pi t}{24}-7\right)\right.\right.\right.\left.+2.4\right)\cdot 0.4\left.\left.\left(\cos\left(\frac{2\pi t}{24}-10\right)+2.6\right)\right)\right)+\left(y_{7}\left(t\right)+c\right)}-k_{1\text{d}}\cdot y_{1}\left(t\right)\\ {\dot{y}}_{2}=k_{2\text{b}}y_{1}\left(t\right)-k_{2\text{d}}y_{2}\left(t\right)-k_{2\text{t}}y_{2}\left(t\right)+k_{3\text{t}}y_{3}\left(t\right)\\ {\dot{y}}_{3}=k_{2\text{t}}y_{2}\left(t\right)-k_{3\text{t}}y_{3}\left(t\right)-k_{3\text{d}}y_{3}\left(t\right)\\ {\dot{y}}_{4}=\frac{\upsilon_{4\text{b}}y_{\text{3}}{\text{(}t\text{)}}^{\text{r}}}{k_{4\text{b}}^{\text{r}}+y_{\text{3}}{\text{(}t\text{)}}^{\text{r}}}-k_{4\text{d}}y_{4}\left(t\right)\\ {\dot{y}}_{5}=k_{5\text{b}}y_{4}\left(t\right)-k_{5\text{d}}y_{5}\left(t\right)-k_{5\text{t}}y_{5}\left(t\right)+k_{6\text{t}}y_{6}\left(t\right)\\ {\dot{y}}_{6}=k_{5\text{t}}y_{5}\left(t\right)-k_{6\text{d}}y_{6}\left(t\right)-k_{6\text{t}}y_{6}\left(t\right)+k_{7\text{a}}y_{7}\left(t\right)-k_{6\text{a}}y_{6}\left(t\right)\\ {\dot{y}}_{7}=k_{\text{6a}}y_{6}\left(t\right)-k_{7\text{a}}y_{7}\left(t\right)-k_{7\text{d}}y_{7}\left(t\right) \end{array}$$

The time *t* is in hours and all concentrations are in nanomolar. *y*_1_ is the concentration of *Per2/Cry* mRNA, *y*_2_ is cysolic PER2/CRY protein complex, *y*_3_ is nuclear PER2/CRY protein complex, *y*_4_ is *Bmal1* mRNA, *y*_5_ is cytosolic BMAL1, *y*_6_ is nuclear BMAL1, and *y*_7_ is nuclear of BMAL1/CLK heterodimer. All parameter values are unchanged from the original model: *v*_1b_ = 9 nM/h, *k*_1b_ = 1 nM, *k*_1i_ = 0.56 nM, *c* = 0.01 nM, *p* = 8, *k*_1d_ = 0.12/h, *k*_2b_ = 0.3 nM/h, *q* = 2, *k*_2d_ = 0.05/h, *k*_2t_ = 0.24/h. *k*_3t_ = 0.02/h, *k*_3d_ = 0.12/h, *v*_4b_ = 3.6 nM/h, *k*_4b_ = 2.16 nM, *r* = 3, *k*_4d_ = 0.75/h, *k*_5b_ = 0.24/h*, k*_5d_ = 0.06/h, *k*_5t_ = 0.45/h, *k*_6t_ = 0.06/h, *k*_6d_ = 0.12/h, *k*_6a_ = 0.09/h, *k*_7a_ = 0.003/h, and *k*_7d_ = 0.09/h.

The differential equations were solved using commercial software, Mathematica 7 (Wolfram Research, Champaign, IL, USA).

## Conflict of Interest Statement

The authors declare that the research was conducted in the absence of any commercial or financial relationships that could be construed as a potential conflict of interest.

## Abbreviations

Dbp, D-binding protein; Dec1, differentiated embryonic chondrocyte-expressed 1; Dec2, differentiated embryonic chondrocyte-expressed 2; E4BP4, E-promoter-binding protein 4; Nr1d1, nuclear receptor subfamily 1, group D; RXR, retinoid X receptor; SCN, suprachiasmatic nuclei; Tef, thyrotroph embryonic factor; ZT, zeitgeber time.
